# Does platform type matter? A semantic analysis of user attitude formation on online platforms

**DOI:** 10.3389/fpsyg.2022.1005429

**Published:** 2022-10-17

**Authors:** Liangbo Zhang, Ge Zhan, Qijing Li, Jifan Ren

**Affiliations:** ^1^School of Economics and Management, Harbin Institute of Technology (Shenzhen), Shenzhen, China; ^2^School of Innovation and Entrepreneurship, Southern University of Science and Technology, Shenzhen, China; ^3^Southampton Business School, University of Southampton, Southampton, United Kingdom

**Keywords:** user attitude, online platform, product and brand perception, semantic analysis, UGC

## Abstract

An online platform is a setting where users may express their attitude in text or visual content. The doctrine thinking in consumer psychology is that greater perceived product value (e.g., more product features or lower price) gives more positive consumer attitude. Because of different types of platforms, however, online users might form their product/brand attitudes in different ways. We gathered 7,264 lines of online reviews about two famous brands on two types of social media platforms: online text-based forums and live-streaming platforms. The data were collected through a web crawler, and semantic analysis was employed to process the data before hypothesis testing. The findings of this study indicate that users’ perception of product features, price levels and brand culture significantly influence user attitude. The more product characteristics communicated on online platforms, the more difficult to formulate a positive user attitude, and users tend to have more positive attitude with higher perceived price. Compared with traditional text-based platforms, contents in live-streaming platforms (e.g., Tik Tok) with less product features, wider culture distance and lower perceived price are favored among users.

## Introduction

Information of traditional online forums is displayed on public platforms with pictures and text as the main content. Over time, online social networking sites have transformed from traditional forums to video-based platforms. Live streaming is a new video platform that can be played, shot and edited through mobile devices, and can be shared in real time and smoothly connected on social media platforms (Wongkitrungrueng and Assarut, 2020). While text-based forum is the public center for communicating among all forum users, live streaming is unique setting where viewers could directly show their attitude with their unique personality and interact with other users (Lin et al., 2021; Zhang et al., 2022).

The most common online settings where users could learn and share product information are text-based platforms (e.g., Twitter, Weibo) and live streaming (e.g., Tik Tok) platforms. The doctrine thinking in consumer psychology is that greater perceived product value, e.g., by adding more product features or lowering down the price, gives more positive consumer attitude (Abedin et al., 2021). We do not take this assumption, given that users of different types of platforms might react to value propositions in different ways and so there should be new lines of thinking or conceptual development on the contingent role played by the type of digital platforms (Wang, 2022). We propose that users of newly developed live-streaming platforms might demonstrate unique tendency and behavior which are different from text-based forum (e.g., Wongkitrungrueng et al., 2020). However, there is still a lack of understanding of the uniqueness of live-streaming platforms and how their users form attitudes towards products and brands in comparison with users of text-based platforms.

Given the significant promotion efforts and marketing expenditures dedicated to digital platforms, this study aims to fill in the knowledge gap by developing a two-dimension conceptual model on the basis of cross platform comparison. This study makes three theoretical contributions. First, this study sheds new light on the important role of platform type as a moderator of the impact of product and brand perception on user attitude. Second, we suggest the contingencies wherein users may value fewer product features, higher perceived price and remote culture distance with a brand. Third, this study addresses an emerging trend in cyber-psychology research relating to users’ formation of attitude on digital platforms.

## Literature

### Live-streaming platform

Recently, live-streaming platforms such as Tik Tok became popular channels for digital communications, and these platforms enable consumers to obtain more information, including both visual and voice information (Liao et al., 2022). A live-streaming platform is a collection of integrated communication tools such as pictures, sound, music, and words (Wang et al., 2021). In order to record their lives, users film videos to record their lives, which are sent to video platforms and caused a surge of views. A video-based platform is like a video diary where users are interested in observing other people’s life, engaging in activities such as learning, communicating, entertaining and experiencing the life experience of others (Bruggeman et al., 2019), and users seek to satisfy psychological needs and form a re-construction of their own cognition (Bruggeman et al., 2019). Live streaming as a way of video socialization has been found an efficient way of visual expression of self (Möller et al., 2020; Prilop et al., 2020).

Digital media is an individualized medium where individuals establish their own networked self-media on the platform, and connect to many others (Möller et al., 2020). It is also a platform to establish well-known influencers who may become a super communicator (Prilop et al., 2020). Usually live streaming adopts a tone that is more suitable for daily life, using formal and informal behaviors and words spreading information to the mass. By commenting with each other, video viewers can quickly and accurately obtain audience suggestions, and can provide timely feedback to these suggestions.

The content of live streaming is the interweaving of reality and performance (Huang et al., 2022). Among the many video platforms, each video viewer may have its own preference, such as Li Ziqi’s county life. Sound is an important element for presenting emotion and creating atmosphere. Live streaming often employs live environmental sounds, such as the sound of rolling waves, the noisy laughter on the street, the background music of the concert, the sound of food chewing from the author, the sound of reading books, and the sound of typing (Wang et al., 2021). These daily themes and sounds are major features that distinguish video platforms from other platforms.

In video platforms, the spread of images is vivid and intuitive. With the support of Internet technology, language and text have realized the function of quickly transmitting information (e.g., Wongkitrungrueng et al., 2020; Wongkitrungrueng and Assarut, 2020; Adjei et al., 2022). In terms of the intuitiveness and vividness of information transmission, the way of image transmission would be more efficient (Wongkitrungrueng et al., 2020). The image is more effective in restoring the scene, the appearance and personality of the characters than text. The use of video media for social interaction, the combination of video background music and editing techniques can effectively convey the thoughts and emotions (Farivar et al., 2021), which are characteristics that text and pictures lack. This is one of the characteristics of video interaction superior to other social interaction on the Internet (Hsu and Chang, 2022). The audience can feel the host’s smile and every sentence and speech during the live broadcast. The real-time dynamics are clearly visible to the audience, and the audience’s knowledge and understanding of the host are perceived more intuitive and true (Evans et al., 2020).

### Product and brand perception on online platforms

Consumer perception of products can be used to indicate users’ brand attitude and purchase intention (Sirgy, 1985; Aaker, 1991; Keller, 1993; Sun et al., 2018; Muda and Hamzah, 2021; Kaur et al., 2022; Kaur et al., 2022). However, off-line settings are dramatically different from digital platforms where users put more emphasis on the ease of use, time efficiency and cost free (Ahn et al., 2004; Asadullah et al., 2018; Majeed et al., 2020). Price is an important source of consumer perceived value (Agarwal and Teas, 2001). It is used to convey the quality and value of products and is among the most important factor influencing consumers’ willingness to buy. Consumers make purchasing decisions not only depending on the quotation of a single store, but also form an expected price in their minds by comparing the prices of products of the same quality. When the price of the alternative product is higher than the expected price, consumers are more likely to perceive fairness. Perceived price has a direct impact on customer satisfaction (Wu et al., 2011; Zeithaml et al., 2018), and the satisfaction can have an impact on their attitude. Usually consumers are more willing to try products with lower prices, but when the price is too low, it will increase perceived risk (Grewal et al., 1994; Wei et al., 2018).

### Platform user attitude

Brand attitude is consisted of the opinion of consumers and emotions toward a brand (Bullmore, 1984). Brand attitude has been defined as a set of consumer’s associations with a brand (Aaker, 1991), and it is related to brand meaning and preserved in memory as a network of associations (Keller, 2003). Consumers are more likely to make purchasing decisions based on favorable brand attitude than the product itself (Bullmore, 1984; Keller, 2003; Graham and Wilder, 2020). When interacting with brands, consumers usually go through a series of stages from week to strong emotions (Fedorikhin et al., 2008). Emotions with brands can be reflected by positive and negative attitudes (Ajzen, 2001; Suh and Youjae, 2006; Gosling and Mason, 2015; Florack et al., 2021). Therefore, consumers’ attitude towards the brand could well measure the strength or depth of the relationship between them (García-de-Frutos and Estrella-Ramón, 2021; Rasool and Pathania, 2021; Zhou and Xue, 2021).

A key driver of brand attitude in e-commerce settings is customer perceived value which is the overall evaluation of the utility of products or services after balancing the perceived benefits of customers with the costs they pay in obtaining products or services (Zeithaml, 1988; Hu and Wise, 2021; Kaur and Singh, 2021). Perceived value is the ratio between perceived gain and perceived loss. Perceived benefits usually relate to physical characteristics, service characteristics and technical support that may be obtained under specific use conditions of the products (Monroe, 1991). Perceived loss includes all costs, transportation, installation, order processing, maintenance and potential failure risks related to the purchase behavior. The essence of customer perceived value therefore is the subjective evaluation value formed by comparing the perceived gains and losses of a product.

**Figure 1:**
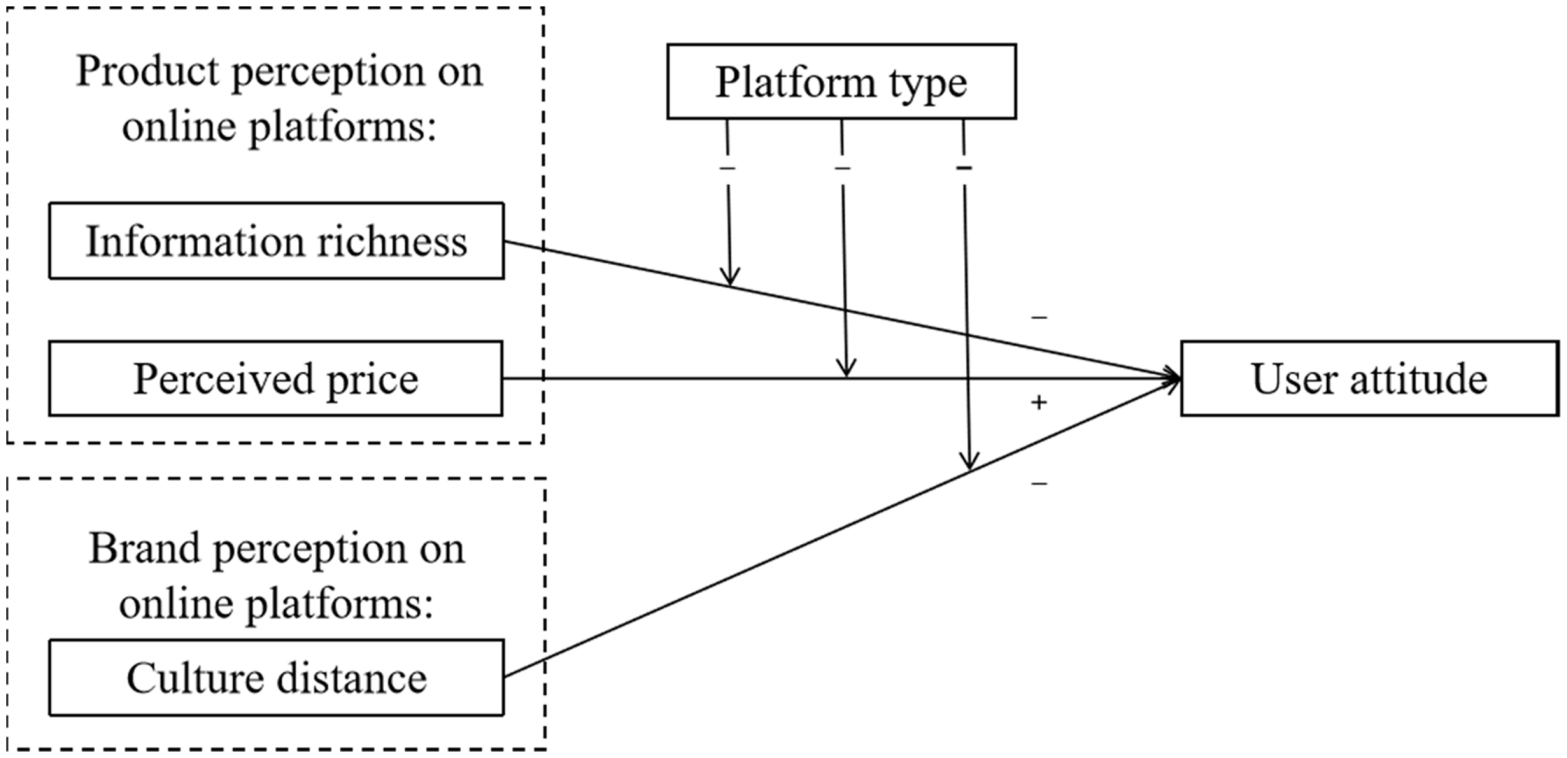
Conceptual framework.

Unlike offline settings where marketers displays products through physical presentation, and customers can directly perceive the value of products by virtue of their sensory organs, e-commerce practitioners use pictures to display products, and customers perceive product value through product pictures and text descriptions (Matosas-López and Romero-Ania, 2021; Souki et al., 2021). Because pictures and text descriptions can be modified, customers cannot directly perceive through the sensory system, and the product value of e-commerce can only be perceived by customers’ subjective feelings (Lopez and Garza, 2021; Rasool and Pathania, 2021). Therefore, traditional logic on the communication of product characteristics and price might be revised, and the new logic should be contingent upon the type of online platforms, given that live streaming demonstrates many unique features compared to traditional online forums. Figure 1 depicts our conceptual model, and this study seeks to answer following research questions:

Are there any significant relationships between product and brand perception on platform user attitude?

Are these relationships contingent upon platform type?

## Materials and methods

As primary data for this study, we gathered various unstructured online reviews about two Chinese brands: Genki Forest and Li Ziqi. While Genki Forest is a popular soft drink brand, Li Ziqi is a well-known food brand in China. First, this study collected data pertaining to these two brands because both brands represented good collection of hedonic and utilitarian products which provide great insights into online review analysis (Zhu et al., 2019). Second, both brands have been posted and commented widely in various digital platforms. The online reviews were collected through a web crawler and came from two types of platforms: traditional online forums and live-streaming platforms. There are many different types of traditional online forums, including large text-based forums like Baidu Tieba, Douban.com, Tianya Forum, Sina.com and Hupu.com, as well as other regional and industrial forums. Live-streaming platforms include platforms such as Tik Tok and Kuaishou. Most digital platform studies so far have drawn conclusions by using Twitter data (Drus and Khalid, 2019). Investigating other digital platforms, particularly newly developed live-streaming platforms, may have good potential to generate new insights into the conceptualization of digital platforms.

The crawling program collected the data from August 31, 2020 to November 21, 2020. After deleting invalid reviews, 7,264 reviews were retained. In late 2020, COVID−19 became a threathening concern widely in many countries. Many consumers shifted their lifestyle dramatically in a way that they would like to spend longer time searching and discussing products online, which provided us an ideal timing and setting to investigate user attitude and behavior in digital platforms.

Before statistically analyzing the online review data, we cleaned up the data with pre-processing, topic classification, and automatic sentiment scoring with Python. User attitude was measured as sentiments towards the two brands, i.e., negative (coded as −1), neutral (coded as 0) and positive (coded as 1). Emotional valence, also known as emotional polarity, refers to the degree of emotional pleasure (Jefferies et al., 2008; Gaspar et al., 2016). The dichotomy of emotional valence divides emotions into two types: positive emotions and negative emotions (e.g., Fredrickson and Branigan, 2005; Allard et al., 2020). Positive attitudes are emotions with positive valence which are affected by internal and external environments, and negative attitudes are emotions with negative valence (Fredrickson and Branigan, 2005; Jefferies et al., 2008).

The meanings of the variables were drawn from online review comments with a semantic analysis approach, which makes use of computers to read and interpret sentences and paragraphs by analyzing text structure and relationships between words. We built a dictionary of keywords, and used R Studio and the Jieba package to tokenize online reviews, and extract keywords. Product and brand concept can be examined and managed from three aspects: functional, symbolic, and experiential (Park et al., 1986). For example, as Genki Forest and Li Ziqi are brands in the food and beverage industry, “experiential” can be captured by keywords such as “delicious” and “flavor.” Information richness was therefore measured as a sum of word frequency of such keywords about the focal product. Similarly, perceived price was measured as a sum of word frequency of keywords (e.g., “cheap,” “expensive,” “luxury,” “good price”, etc.) reflecting lower or higher price.

Culture distance was measured by the location-brand culture consistency. For example, spicy foods are favored by residents living in locations such as Chongqing, Sichuan, and Hunan in China. Li Ziqi’s foods are mostly spicy, so consumers living in these locations may find culture closeness from consuming Li Ziqi. Hence, the psychic distance between users and brand culture was employed to measure culture distance (0 for smaller distance, 1 for bigger distance).

Platform type was measured in binary nominal values (0 for live-streaming platforms, 1 for online text-based forums). Investigating the nuances of these different types of platforms may contribute to the understanding on the concept of digital platforms (Asadullah et al., 2018). Consistent with prior studies (Coussement and Van den Poel, 2008), we used key term frequency to measure the independent variables (information richness and perceived price).

## Results

Table 1 presents the demographic characteristics of the sample. The sample had approximately same proportion of traditional text-based platform users (51.7%) and live-streaming platform users (48.3%). The average user attitude was positive emotion and close to 0.3 which might reflect the case that COVID-19, although affecting life and economy badly, had been controlled effectively in China during the data collection period. Table 2 shows the correlation coefficients among user attitude and all other factors. VIF values among the variables ranged from 1.05 to 1.55, showing that multicollinearity is not a serious problem.

**Table 1:** Demographic characteristics.

Variable	*N*	Mean	Std. Dev.	Min	Max
User attitude	7,264	0.296	0.613	−1	1
Information richness	7,264	4.241	3.877	1	36
Perceived price	7,264	−0.350	2.345	−38	10
Culture distance	7,264	0.684	0.465	0	1
Platform type	7,264	0.517	0.500	0	1

**Table 2:** Correlation matrix.

	User attitude	Information richness	Perceived price
User attitude	1.000		
Information richness	−0.067	1.000	
Perceived price	0.039	−0.119	1.000
Culture distance	−0.092	−0.209	−0.075

Table 3 presents the results of regression models. In Table 3, model 1 showed the main effects of the three components of product and brand perception on online platforms. Both information richness (*β* = −0.077, *p* < 0.001) and perceived price (*β* = 0.006, *p* < 0.05) were significantly associated with user attitude. Culture distance was negatively and significantly related to user attitude (*β* = −0.140, *p* < 0.001). As shown in Table 3, the moderation effect of platform type on the relationship between information richness and user attitude was significant and negative (*β* = −0.087, *p* < 0.001). Platform type indicated negative moderating effects on the impact of perceived price (*β* = −0.047, *p* < 0.001) and culture distance (*β* = −0.159, *p* < 0.001) on user attitude.

**Table 3:** Effects on user attitude.

	Model 1	Model 2	Model 3	Model 4
(Constant)	0.353 (0.022)***	0.280 (0.025)***	0.310 (0.023)***	0.355 (0.022)***
ln(Information richness)	−0.077 (0.014)***	−0.068 (0.014)***	−0.011 (0.016)	−0.078 (0.014)***
ln(perceived price)	0.006 (0.003)*	0.048 (0.007)***	0.002 (0.003)	0.006 (0.003)
Culture distance	−0.140 (0.016)***	−0.203 (0.019)***	−0.219 (0.019)***	0.009 (0.053)
Platform type×information richness			−0.087 (0.011)***	
Platform type×perceived price		−0.047 (0.007)***		
Platform type×culture distance				−0.159 (0.054)**
*N*	7,264	7,264	7,264	7,264
*F*	33.95***	36.09***	40.21***	27.67***

*p < 0.05; **p < 0.01; ***p < 0.001.

## Discussion

### Theoretical implications

The results reveal that the more product characteristics communicated on online platforms, the more difficult to formulate a favourite user attitude. This finding provides more evidence on the case that greater product detail given in longer reviews does not always add to perceived helpfulness of the review. As indicated by a recent study of online customer reviews on 12 products from four categories (Jia et al., 2022), longer reviews are more likely to yield greater helpfulness only for think products. Online contents with sophisticated product features would make it difficult for viewers to understand and give comments online. Rather, they may value more straight forward and neatly framed product information.

Product price is one of the most important factors consumers consider when making a purchase decision (Wang et al., 2022). Interestingly, this study shows that users tend to have more positive attitude with higher perceived price, which is different from previous findings on the effect of price on user attitudes in traditional shopping settings (Chao and Liao, 2016). In digital settings which are characterized by an enormous among of uncertainties and unknown others, the negative impact of low price may dominate consumers’ mind, and so they may formulate unfavourable attitude. The results of this study confirm the view that low price is not always favored by consumers. Users in online platforms would have greater trust in the quality of the product or brand with higher perceived price.

Previous research suggests that patterns and drivers of consumer pre-purchase activities, purchase decisions, and post-purchase commitment may vary by culture (Shavitt and Barnes, 2020). According to the results, lower culture distance suggests more favorable user attitude. Locals can easily use the quality of the best or their favorite local products to judge the offerings from the brands on the Internet. People who are familiar with local products, are more likely to appreciate these brands. It is natural that people are more willing to talk and spread the information that they are familiar with. This finding can also be explained with He et al.’s (2021) three forms of brand-owned SMCM, i.e., conversation, storytelling and customer interaction. Culture closeness gives more opportunities for all these three forms of social-media content marketing. Furthermore, the more consistent the brand personality and the consumer’s self-concept of a product or brand, the easier it is for consumers to choose and favor.

### Practical implications

Although information disclosure about products in online platforms may have an important impact on user attitude (Xu et al., 2021) the more aspects consumers reveal about product characteristics, the more difficult it is for the products to meet consumer expectations. Therefore, we recommend that practitioners to make good effort to neatly design and define product notions. The primary understanding of product characteristics held by customers is usually accompanied by distinctive expectations that are easy to perceive and measure, as well as features that are clear and concrete that can be used to judge the product’s performance easily. The situation is even riskier considering that different customers would hold different understanding. In the effort of combining several related characteristics into one notion, the companies could unify and soften the separated characteristics, and protect the brand from severe judgment. Meanwhile, such a shift in perception aids in the transformation of a clear expectation into curiosity and desire, lowering the likelihood of dissatisfaction. In fact, such implementation can be observed in big companies, such as Apple promoting “Retina Display” over the years, and Intel promoting the “EVO” standard since 2020.

Even though online environment is a virtual setting, the online contents would exert a strong influence on the performance of the company because consumers would search for and read those online contents that are publicly available and easily accessible. For live-streaming platforms, UGCs with less product features, distant culture and lower perceived price are favored among users. As a new application form in the era of social media, visual communication based on live broadcasts and short videos has greatly promoted self-expression, and has also given new characteristics and connotations to para-social interactions (Farivar et al., 2021; Pera et al., 2022). In essence, para-social relationships are non-reciprocal. Compared to forums, which usually only contain text and pictures, video platforms provide more dimensions to judge from, including sound, movement of objects, and a full record of procedures that last for longer time. Videos also provide more elements that make sharing behaviors meaningful than simple text and attitudes.

As a video-sharing website, Bilibili.com has become an important para-social platform in China. It allows users to post comments while watching the video, and their comments appear on the screen immediately and are played in synchronization with the video. The comments posted by users while watching a video are an instant response, that is, quasi-social interaction (Peters et al., 2018; Pera et al., 2022). The para-social short video platform has changed the way audiences receive information, convey emotions, and interact with each other, as this new type of digital platform is characterized by life-oriented, real-time, socialized, and interactive (Stewart et al., 2019; You et al., 2020). However, the entertaining and fragmented cultural atmosphere of the social short video platform has also given birth to a digital ecology with a high degree of media dependence (Park et al., 2018; Jia et al., 2022). In this new digital environment where the audience is overly dependent on the media, the discourse power of elite culture is gradually disintegrated, and the critical consciousness of the audience would be gradually dispelled. As video platforms, compared to traditional online forum, represent more para-social, emotional and entertaining relationships, users may not emphasize more product features or culture closeness, rather they may give higher weight on more affordable price.

## Conclusion

The semantic meanings of online UGCs have undeniable influences on consumer decision-making and purchasing behaviors. However, due to technological limitations and the complexity of the Chinese language, large-scale analysis of the semantic meanings of online content in China has only recently become possible. Based on semantic analysis of online comments, this study attempts to investigate the factors that influence online user attitude. This study uncovered several significant trends regarding online platforms.

Textual and semantic analytics are still in infant stage. Despite the fact that we had found many different libraries of stop words in this study, the final tokens performances are less than satisfactory. We decided combining the libraries, and include some recent buzzwords. Research on video platforms should not only focus on the technical aspects, but also need to analyze psychological and social factors. Future studies may examine other types of product-consumer consistency, such as individual-overall rating (Jia et al., 2022), self-image, brand-image consistency (Deng et al., 2021). As COVID-19 has stimulated more online consumption and communication, the impact of COVID-19 on digital platforms should be a prominent research question in future cyberpsychology studies.

## Data availability statement

The original contributions presented in the study are included in the article, further inquiries can be directed to the corresponding author.

## Author contributions

LZ, GZ, JR, and QL were involved in the study conceptualization, design, and implementation. JR and GZ obtained the funding. LZ, GZ, and QL were involved in the data analysis and in interpreting the data. LZ and GZ wrote the first draft. LZ and JR contributed to visualizing and supervising the research. All authors contributed to the article and approved the submitted version.

## Funding

This research was funded by the Natural Science Foundation of China (grant numbers 71831005 and 71832015), Shenzhen Humanities & Social Sciences Key Research Bases (grant number KP191001), Higher Education Enhancement Plan by the Guangdong Education Department (UICR0400011-21), UIC Research Grant (grant no. R202027), and Joint Research Project from the Guangdong Planning Office of Philosophy and Social Science (GD20XGL55).

## Conflict of interest

The authors declare that the research was conducted in the absence of any commercial or financial relationships that could be construed as a potential conflict of interest.

## Publisher’s note

All claims expressed in this article are solely those of the authors and do not necessarily represent those of their affiliated organizations, or those of the publisher, the editors and the reviewers. Any product that may be evaluated in this article, or claim that may be made by its manufacturer, is not guaranteed or endorsed by the publisher.
